# Consensus of Experts on the Treatment of Sexual Dysfunction after Surgery for Prostate Cancer in Taiwan

**DOI:** 10.3390/jcm12030740

**Published:** 2023-01-17

**Authors:** Yu Chen, Hong-Chiang Chang, William J. Huang, Chii-Jye Wang, Thomas I-Sheng Hwang, Chun-Hou Liao, Chia-Chu Liu, See-Tong Pang, Eric Yi-Hsiu Huang, Chih-Wei Tsao, Kuo-Chiang Chen, Shih-Ping Liu, Chao-Yuan Huang, Cheng-Hsing Hsieh, Bang-Ping Jiann

**Affiliations:** 1 Department of Surgery, Division of Urology, Linkou Chang Gung Memorial Hospital, College of Medicine, Chang Gung University, Taoyuan 333, Taiwan; 2 School of Medicine, National Tsing Hua University, Hsinchu 300044, Taiwan; 3 Department of Surgery, Division of Urology, Far-Eastern Memorial Hospital, New Taipei City 220, Taiwan; 4 Department of Urology, Taipei Veterans General Hospital, Taipei 112, Taiwan; 5 Department of Urology, E-Da Dachang Hospital, Kaohsiung 82446, Taiwan; 6 School of Medicine, Fu-Jen Catholic University, New Taipei City 242062, Taiwan; 7 Department of Surgery, Shin Kong Memorial Hospital, New Taipei City 111045, Taiwan; 8 Department of Urology, Cardinal Tien Hospital, Fu-Jen Catholic University, New Taipei 362, Taiwan; 9 Kaohsiung Medical University Hospital, Kaohsiung Medical University, Kaohsiung 80756, Taiwan; 10 Urology Division, Taipei Veterans General Hospital, Taipei City 112, Taiwan; 11 Department of Surgery, Division of Urology, Tri-Service General Hospital, National Defense Medical Center, Taipei 11490, Taiwan; 12 Cathay General Hospital, Fu Jen Catholic University, Taipei City 106, Taiwan; 13 Department of Urology, National Taiwan University Hospital, College of Medicine, National Taiwan University, Taipei 11031, Taiwan; 14 Division of Urology, Buddhist Tzu-Chi General Hospital Taipei Branch, School of Medicine, Buddhist Tzu-Chi University, Hualien City 970, Taiwan; 15 Division of Urology, Department of Surgery, Kaohsiung Veterans General Hospital, Kaohsiung 813414, Taiwan

**Keywords:** prostate cancer, sexual dysfunction, Taiwan, erectile dysfunction

## Abstract

According to the Taiwan Cancer Report, in 2018, prostate cancer was one of the top five cancers reported in men. Each year, many patients with prostate cancer undergo radical prostatectomy (RP) therapy. One of the most common RP complications is erectile dysfunction (ED). Although consensus guidelines for the management of sexual dysfunction after prostate cancer surgery have been developed for many Western and Asian countries, no such clinical practice guidelines have been developed for Taiwan. The consensus opinions expressed in this article were discussed by numerous experienced physicians in Taiwan, based on both existing international guidelines and their individual experiences with clinical trials and providing advice to clinical physicians on how to inform patients of the risk of ED prior to surgery. This review also discusses how recovery and rehabilitation may be affected by socioeconomic status, the existence of an intimate relationship, comorbidities, or the need for cancer adjuvant therapy and how to determine rehabilitation goals and provide appropriate treatments to assist in the recovery of both short- and long-term sexual function.

## 1. Expert Consensus Were Reached by Face-to-Face Meetings

This expert consensus was reached through two face-to-face meetings, and agreements were reached by anonymous voting. The experts were recruited mostly from the Taiwanese Association of Andrology through surveys. Others, who were also recruited through the same surveys, were considered key-opinion-leaders regarding erectile dysfunction in Taiwan. 

During the two face-to-face meetings, up-to-date evidence was reviewed by experts. Built upon the evidence, experts discussed their opinions as well as practicing experiences in Taiwan. The consensus was stated and revised accordingly to specific practical scenarios in Taiwan afterward. At the final part of the meetings, anonymous voting was held. Only those agreements reached above 80% were listed as experts’ consensus in Taiwan.

## 2. Radical Prostatectomy Results in Erectile Dysfunction in 30–50% of Patients

Radical prostatectomy (RP) is the most common clinical treatment for prostate cancer and is associated with various complications, including erectile dysfunction (ED), anejaculation, difficulty with orgasm, painful ejaculation, urinary incontinence during orgasm, and changes in penis length or curvature. A study showed that among patients who experience postoperative ED, these complications could persist for two years [1]. Among patients who do not undergo penile rehabilitation, ED is the most common complication reported. No differences in the complication rate have been observed according to the surgical approach, with similar rates reported for RPs performed using open, laparoscopic, and robot-assisted laparoscopic techniques. The incidence of ED is 30–50%, even among patients who undergo nerve-sparing RP (NSRP) [2]. Therefore, most patients will require treatment for sexual dysfunction after prostate cancer surgery.

## 3. Evaluating Preoperative Sexual Function and Informing Patients of Potential Postoperative Risks

During RP, damage to the arteries and the cavernous nerve can lead to corporal hypoxia, increasing the production of reactive oxygen species, transforming growth factor (TGF)-β1, and cytokines, inducing apoptosis in smooth muscle cells and the development of corporal fibrosis. The incidence of ED may be associated with age, preoperative erectile function (EF), disease severity, and the surgeon’s experience in performing RP. Patients without vascular-related comorbidities who are younger than 60 years and who receive medication therapy within six months of RP may experience better recovery than patients in other groups [3].

According to a preoperative survey, only 30–40% of patients are willing to undergo EF rehabilitation, and the proportion of patients willing to engage in rehabilitation is even smaller among patients 70 years or older. Postoperative rehabilitation not only improves sexual function recovery but also prevents the development of fibrosis and maintains penis size, which can lead to improved quality of life. Patients should be provided with multidisciplinary information to encourage their willingness to engage in EF rehabilitation following RP.

## 4. IIEF Questionnaire as a Diagnostic Tool for Assessing EF

We should inform patients regarding the risks of ED associated with various prostate cancer therapies, including surgery, high-intensity focused ultrasound, and radiotherapy. The International Index of Erectile Function (IIEF) is a simple diagnostic tool used to study the prevalence of ED. By administering this questionnaire prior to therapy, we can estimate the likelihood of recovery and provide patients with an appropriate prognostic prediction. A good prognosis requires the selection of appropriate treatment. Using the IIEF to evaluate pre-treatment EF (IIEF-EF) prior to performing RP could prevent unrealistic expectations for postoperative recovery. Furthermore, some experts have suggested using the IIEF to assess EF during regular clinical evaluations of ED.

## 5. Sexual Recovery after Prostate Cancer Requires Multidisciplinary Care

After surgery, patients should be monitored for tumor control status and urinary incontinence, and the recovery plan should be adjusted accordingly. However, the management of ED following prostate cancer therapy would increase the workload of oncology physicians. Including urology physicians in the management of post-treatment ED recovery would reduce the load among oncology physicians. Some experts in Taiwan recommended the use of the term “recovery” rather than “rehabilitation” because ED is a common physical condition after operation. In a retrospective study, the rehabilitation outcomes of 92 patients were examined following prostate cancer surgery. The results showed that patients who initiated rehabilitation longer than six months after RP experienced a 1.8-fold increase in the risk that sexual function would not recover (relative risk [RR] = 2.8, *p* < 0.01) compared with patients who initiated rehabilitation sooner [3]. Mulhall et al. reported that patients who initiated rehabilitation within six months after RP scored higher on the IIEF for EF than those who initiated rehabilitation after six months (22 vs. 16, *p* < 0.001), [4] further highlighting the importance of early rehabilitation.

Multidisciplinary care should always be taken into consideration. There are several underlying diseases which may cause ED, such as Peyronie’s disease [5]. Underlying diseases, such as diabetic mellitus [6], may also affect the treatments of ED. With the help of multidisciplinary experts, both ED and underlying diseases can be better managed.

## 6. PDE5-Is Re First-Line Treatments for Post-RP EF Rehabilitation

Phosphodiesterase phosphate-5 inhibitors (PDE5-Is) are the most commonly recommended first-line therapeutic option for ED, including ED post-RP. Many treatment options exist for post-RP EF rehabilitation, which can be categorized as invasive and non-invasive interventions. Most clinicians opt for non-invasive first-line treatments, including PDE5-Is and vacuum erection devices (VEDs). If first-line treatments are not effective, more invasive options, such as intracavernosal injection (ICI) and transurethral prostaglandin E1 (PGE1) injections, may be elected. If both first- and second-line treatments are ineffective, a penile prosthesis may be considered. Many new treatment options have been developed, such as low-intensity extracorporeal shockwave therapy (LiESWT), platelet-rich plasma (PRP), and intracavernosal stem cell therapy [7,8,9,10]. Thus far, LiESWT has become the first-line treatment choice recommended by the European Association of Urology (EAU). However, the treatment guidelines published by the American Urological Association (AUA) indicate that LiESWT, PRP, and intracavernosal stem cell therapy continue to require additional support from clinical data (Table 1) [7,8]. The 2019 EAU guidelines also recommended that PDE5-Is could be used as a first-line treatment among patients who received bilateral NSRP (BNSRP) [7]. PDE5-Is have many benefits, including high efficacy, ease of use, and good safety profiles, and PDE5-Is are generally well-tolerated and improve quality of life [7]. Other guidelines from other associations, including the AUA, the Korean Society for Sexual Medicine and Andrology (KSSMA), and the Japanese Society for Sexual Medicine (JSSM), recommend the use of PDE5-Is as a first-line treatment after RP (Table 1).

One survey of AUA members on the management of patients following RP showed that the PDE5-Is are the most common first-line treatment option for EF rehabilitation, followed by VED, ICI, and transurethral PGE1 administration. Half of patients wait to initiate rehabilitation until after catheter removal, whereas 32% of patients initiate rehabilitation within four months after RP, and only 11% of patients initiate rehabilitation immediately after RP. In addition, 45% of patients discontinue rehabilitation after 12–18 months, whereas 25% of patients discontinue rehabilitation within 12 months [11].

The conditions in Taiwan are similar to those reported in America. Patients often discontinue treatment early, and only 50% of patients continue treatment beyond the first year. Major reasons provided for PDE5-I discontinuation included high costs and unsatisfactory treatment outcomes. Physicians should attempt to reach a consensus with their patients and help patients choose the most appropriate and cost-effective treatment for reaching their rehabilitation goals. If patients express dissatisfaction with treatment outcomes, we suggest that physicians attempt to encourage treatment continuation. Long-term EF rehabilitation can be very expensive. However, PDE5-Is can be an effective first-line treatment option.

## 7. PDE5-I Combined with Libido Stimulation May Result in Better Recovery

In a meta-analysis comparing IIEF scores among patients receiving NSRP, PDE5-Is were found to be more beneficial than placebo for improving EF after RP [12]. In trials of the PDE5-Is sildenafil, vardenafil, and tadalafil, nightly use was compared with on-demand use. The average IIEF-EF did not differ significantly between nightly and on-demand use of sildenafil. However, the proportion of patients with IIEF-EF scores >21 was larger among patients in the on-demand group than in the nightly use group [13]. In trials of vardenafil, on-demand use resulted in higher successful intercourse rates than nightly use [14]. However, daily vardenafil use resulted in higher IIEF-EF scores than on-demand use [15]. A meta-analysis revealed that compared with on-demand use, regular use (three times per week) was more effective for improving ED [12]. However, the frequency of medication use is generally accepted as not being the most important factor for determining treatment success. Instead, libido stimulation appears to have a strong effect on PDE5-I efficacy. Therefore, treatment success is likely to be determined by libido stimulation rather than medication frequency. People in Taiwan have been reported to engage in sexual intercourse 1.5 times per week, on average. Therefore, treatments may be more effective if patients are recommended to use PDE5-Is intermittently, 2–3 times per week, combined with libido stimulation. Successful sexual intercourse is not necessary. If patients experience sexual desire with high frequency, the medication frequency may be increased, or patients can be switched to tadalafil, which has a longer effect.

A standard dose of PDE5-Is (100 mg sildenafil; 20 mg tadalafil, or 20 mg vardenafil) appears to result in the best efficacy; however, patients’ economic status and their needs for sexual intercourse should also be considered. A lower dose (50 mg sildenafil or 5 mg tadalafil) can be used by patients without sexual needs, and the dose can later be increased to the standard dose when sexual needs arise. For example, patients using low-dose sildenafil can be moved to the 100 mg dose. Another option is the use of a second low-dose PDE5-I, such as 5 mg tadalafil, for daily use, with 100 mg sildenafil used prior to sexual intercourse.

## 8. PDE5-Is Are Selected Based on Treatment Duration, Safety, Cost, and Patients’ Willingness

Sildenafil, vardenafil, and tadalafil have all been approved for use in Taiwan. When choosing PDE5-Is, various factors should be considered, including treatment duration, safety, cost, patients’ willingness to use the treatment, and physicians’ clinical experiences with the treatment. Sildenafil is supported by the most clinical evidence, and most patients demonstrate good tolerance. According to pharmacokinetic data (Table 2), tadalafil has a longer duration of activity, which may result in better efficacy for morning erections. However, tadalafil is also associated with unpleased side effects, including myalgia and dyspepsia. Vardenafil has a rapid onset and can be used with a shorter lead time before sexual activity but should be avoided in patients with heart disease.

Some experts indicated that no standards exist for choosing medicine, as the option depends on multiple factors, including the environment, the patient’s physical and psychological status, and the operation conditions. Medications should be considered immediately after ED is reported.

## 9. Sildenafil Is Better Than Other PDE5-Is for Increasing Oxygen Uptake

The major concept underlying penile rehabilitation strategies after RP is the preservation of adequate oxygenation. The earlier establishment of adequate oxygenation is associated with a greater likelihood of achieving normal EF [16]. A preclinical study showed that PDE5-I use could increase intracavernous pressure and blood flow, contributing to EF recovery [17].

A study was conducted to compare PDE5-I efficacy, which showed no differences in IIEF-EF scores among the various PDE5-Is. However, sildenafil improved peak systolic velocity (PSV) more than the other PDE5-Is (Table 3) [18]. This finding indicates that sildenafil represents a better treatment option for improving penile blood flow in the long term. Moreover, studies related to pulmonary arterial hypertension also showed that sildenafil is more effective than other PDE5-Is for increasing pulmonary arterial oxygenation [19]. In summary, because the major goal of penile rehabilitation after RP is the preservation of adequate oxygenation, sildenafil may be more effective than other PDE5-Is for long-term EF.

## 10. Sildenafil Treatment for ED after RP Should Begin Immediately

Among examined first-line ED treatments following RP, sildenafil had the lowest withdrawal rates and sufficient evidence of efficacy [20]. In patients who initiated sildenafil within six months after RP, 58% experienced both unassisted erections and PDE5-I–assisted erections by 18 months after RP. Among patients who initiated sildenafil more than six months after RP, only 30% recovered EF, demonstrating the importance of early treatment initiation [21].

Among patients who underwent BNSRP, followed by sildenafil treatment, 76% reported the ability to engage in successful vaginal intercourse. Among patients who underwent unilateral NSRP and non-NSRP, followed by sildenafil treatment, 53.5% and 14.2%, respectively, reported the ability to engage in successful vaginal intercourse [22]. Other prognostic factors include EF before RP, age, and the interval between RP and sildenafil initiation [22]. Studies have shown that PDE5-Is have clinical benefits in all types of patients, with the most significant improvements observed in patients with moderate ED risk [23].

## 11. Patients Who Do Not Experience Satisfactory Outcomes with PDE5-Is, Combination Therapy May Be Started Immediately

Compared with VED monotherapy, VED combined with sildenafil resulted in better penile rigidity and sexual satisfaction [24]. Moreover, the combination therapy was also able to reduce the time to EF recovery [25]. In addition, the early initiation of combination PDE5-I plus ICI may contribute to reduced ICI dosage, decreasing penile discomfort, and increasing successful sexual activity [26].

PDE5-I monotherapy was recommended to be extended from 6 months to 1 year. However, when PDE5-I monotherapy has a poor effect within the first 1–4 months, the combination of PDE5-I with VED, ICI, or PGE1 therapy may be considered to increase patients’ confidence and satisfaction. The most common combination therapy options include maintaining oral medication with the additional use of ICI or VED. Although studies have shown that both ICI and VED combination therapy have equivalent efficacy, VED has higher costs and requires longer treatment times, with variable efficacy among patients. By contrast, ICI can be administered at follow-up visits, can be interrupted at any time, and does not require consideration of postoperative neurological status. Therefore, most physicians choose PDE5-I plus ICI combination therapy as the first choice. Combination therapies involving PDE5-I plus a medicated urethral system for erection, LiESWT, or PRP require the collection of additional clinical data to demonstrate their efficacy.

## 12. Conclusions

Patients’ rehabilitation goals, expectations, motivations, socioeconomic status, intimate relationships, comorbidities, and requirements for cancer adjuvant therapy should be considered when selecting appropriate treatment options for post-RP EF rehabilitation. Various treatment options are available for EF rehabilitation, including both invasive and non-invasive interventions. Multidisciplinary care could be useful for improving EF. When discussing potential future treatments, physicians should provide realistic treatment options and outcomes and choose a treatment that best matches the patient’s rehabilitation goals.

Daily PDE5-I use was not found to be more effective than regular intermittent use for improving ED after RP. However, libido stimulation is a key factor affecting PDE5-I outcomes. Therefore, regular use of PDE5-Is plus sexual activity 2–4 times per week may be the most appropriate therapy. Because a major goal of penile rehabilitation after RP is the preservation of adequate oxygenation, evidence suggests that sildenafil may be more effective than vardenafil and tadalafil for long-term therapeutic outcomes.

## Figures and Tables

**Table 1:** Treatment recommendation for post-RP erectile function rehabilitation.

	EAU 2019 [7]	AUA 2018 [10]	KSSMA 2013 [9]	JSSM 2008 [8]
First-line	PDE5-I, VED, LiESWT	PDE5-I, VED	PDE5-I, VED	PDE5-I
Second-line	ICI, transurethral PGE1	ICI, transurethral PGE1	ICI	VED, ICI
Third-line	Penile prostheses	Penile prostheses	Penile prostheses	Penile prostheses
Conditional recommendation *		LiESWT, PRP, intracavernosal stem cell therapy		

* Conditional recommendation, or expert opinion, should be considered investigational or experimental therapy. AUA, American Urological Association; EAU, European Association of Urology; ICI, intracavernosal injection; JSSM, Japanese Society for Sexual Medicine; KSSMA, Korean Society for Sexual Medicine and Andrology; LiESWT, low-intensity extracorporeal shockwave therapy; PDE5-I, phosphodiesterase type 5 inhibitor; PGE1, prostaglandin E1; PRP, platelet-rich plasma; VED, vacuum erection device.

**Table 2:** Pharmacokinetic data for the three PDE5-Is [9].

Parameter	Sildenafil 100 mg	Tadalafil 20 mg	Vardenafil 20 mg
T_max_ (h)	0.8–1	2	0.9
T_1/2_ (h)	2.6–3.7	17.5	3.9
Action duration (h)	0.5–4	1–36	0.5–5
C_max_ (µg/L)	560	378	18.7
AUC (µg/h/L)	1685	8066	56.8
Protein binding (%)	96	94	94
Bioavailability (%)	41	NA	15

AUC, area under the curve; C_max_, maximum plasma concentration; h, hour; NA, not available; PDE5-Is, phosphodiesterase type 5 inhibitors; T_1/2_, terminal half-life; T_max_, time to maximum plasma concentration.

**Table 3:** Average IIEF and PSV after each treatment [18].

Characteristics	Sildenafil 50 mg	Sildenafil 100 mg	Tadalafil 20 mg	Vardenafil 20 mg	*p*-Value
IIEF questions 1–5 (score, 95% CI)	18.3 (17.6–20.2)	18.8 (17.1–25.6)	17.0 (15.5–20.1)	17.9 (15.1–20.8)	0.493
PSV (cm/s, 95% CI)	46.5 * (41.2–51.7)	46.9 * (41.3–52.4)	35.8 (32.9–38.6)	37.3 (31.6–43.1)	0.001

* Significant improvement compared with tadalafil and vardenafil. CI, confidence interval; IIEF, International Index of Erectile Dysfunction; PSV, peak systolic velocity.

## Data Availability

No new data were created or analyzed in this study. Data sharing is not applicable to this article.
